# Fostering cognitive strategies for learning with 360° videos in history education contexts

**DOI:** 10.1007/s42010-022-00154-x

**Published:** 2022-08-04

**Authors:** Valentina Nachtigall, Selina Yek, Elena Lewers, Christian Brunnenberg, Nikol Rummel

**Affiliations:** 1 Lehrstuhl für Pädagogische Psychologie und Bildungstechnologie, Institut für Erziehungswissenschaft, Ruhr-Universität Bochum, Universitätsstr. 150, 44801 Bochum, Germany; 2 Didaktik der Geschichte und Public History, Historisches Institut, Ruhr-Universität Bochum, Universitätsstr. 150, 44801 Bochum, Germany

**Keywords:** Virtual reality, 360° videos, History education, Self-regulated learning, Cognitive strategies, Virtual Reality, 360° Videos, Geschichtsvermittlung, Selbstreguliertes Lernen, Kognitive Lernstrategien

## Abstract

Learning settings in and out of school are increasingly relying on the use of virtual reality applications, such as 360° videos, to make learning an exciting and vivid experience for students. This applies especially to history-learning contexts. Learning with immersive representations of history-related contents requires a critical examination and reflective processing of the learning content. Cognitive strategies, such as organizing and elaborating information correspond with competencies which are assumed to be important for students’ critical examination and reflective processing of history-related content. Research on self-regulated learning (SRL) suggests that the use of cognitive strategies can be promoted through respective SRL trainings. Thus, in the present quasi-experimental study (*N* = 164), we investigated the effectiveness of a SRL training, which adds to regular instruction on processing history-related learning materials, for students’ use of cognitive strategies when examining immersive history-related 360° videos. Our results show that students who practiced analyzing 360° videos within an explicit SRL training used more cognitive strategies than students who received an implicit SRL training on how to analyze these videos. Further findings suggest that the use of these cognitive strategies probably helped students of the training condition (explicit SRL training) to make less imprecise or trivial analyses and to draw more reflective conclusions than students of the control condition (implicit SRL training). By combining research on SRL and history education, our study may provide a new impulse for empirical research on competence-oriented learning with history-related virtual reality media.

## Abstract (de)

Um Schüler*innen interessante und anschauliche Lernerfahrungen zu ermöglichen, finden Virtual Reality Anwendungen, wie 360° Videos, zunehmend an Bedeutung für Lernumgebungen innerhalb und außerhalb der Schule. Eine zunehmende Nutzung dieser Anwendungen ist insbesondere in Kontexten der Geschichtsvermittlung zu verzeichnen. Das Lernen mit immersiven Repräsentationen geschichtsbezogener Inhalte verlangt jedoch eine kritische und reflektierte Auseinandersetzung mit den dargestellten Inhalten. Kognitive Strategien, wie das Organisieren und Elaborieren von Informationen korrespondieren mit Kompetenzen, die für eine reflektierte und kritische Auseinandersetzung mit geschichtsbezogenen Inhalten als wichtig erachtet werden. Forschungsbefunde zum selbstregulierten Lernen (SRL) weisen darauf hin, dass SRL Trainings die Nutzung kognitiver Lernstrategien entsprechend fördern können. Vor diesem Hintergrund wurde in der vorliegenden quasi-experimentellen Studie (*N* = 164) die Effektivität eines SRL Trainings, welches reguläre Unterrichtsmethoden zur Auseinandersetzung mit geschichtsbezogenen Materialien ergänzt, für die Nutzung kognitiver Strategien beim Analysieren immersiver geschichtsbezogener 360° Videos untersucht. Die Ergebnisse zeigen, dass Schüler*innen, welche mithilfe eines expliziten SRL Trainings die Analyse der Videos übten, mehr kognitive Strategien für die Videoanalysen nutzten als Schüler*innen, die nur ein implizites SRL Training erhielten. Weitere Befunde deuten darauf hin, dass die vermehrte Nutzung kognitiver Strategien die Schüler*innen der Trainingsbedingung (explizites SRL Training) dabei unterstützte, weniger unpräzise oder banale Analysen durchzuführen sowie mehr reflektierte Schlussfolgerungen zu ziehen als es in der Kontrollbedingung (implizites SRL Training) der Fall war. Durch die Verknüpfung geschichtsdidaktischer Forschung mit Forschungsbefunden zum selbstregulierten Lernen setzt die vorliegende Studie neue Impulse für die empirische Erforschung kompetenzorientierten Lernens mit geschichtsbezogenen Virtual Reality Medien.

## Virtual reality in history education contexts

In recent years, there has been an increased interest in the use of virtual reality (VR) in different educational contexts in and out of school (Black 2017; Liu et al. 2017; Di Natale et al. 2020). This increasing use of VR media as educational technologies can also be observed in the field of history education, where VR promises to represent the past and to travel in time (Bunnenberg 2018), and is even considered being a part of novel, exciting, and promising learning and teaching approaches for school (Schulministerium NRW 2021).

VR media can be more or less immersive (Di Natale et al. 2020) and are usually designed with the goal of enabling perceived presence in a virtual space (Cummings and Bailenson 2015). VR applications with head-mounted displays are usually considered as highly immersive as they enable the users to move within the virtual environment (i.e., high level of tracking), take up the user’s entire field of view, provide binocular vision, and enable the users to experience the virtual world from a first-person perspective (Di Natale et al. 2020; Cummings and Bailenson 2015). Less immersive VR applications use conventional computers (Hartmann and Bannert 2022) and deliver the experiences of the virtual world merely through a desktop (Di Natale et al. 2020). Consequently, these applications provide only monoscopic vision and enable users either to only observe movements within the virtual space or manipulate these moves by using a mouse or joystick but not by moving their eyes, head, or body (i.e., low level of tracking) (Di Natale et al. 2020; Cummings and Bailenson 2015).

Both highly immersive as well as less immersive VR media are used as educational technologies in history-learning contexts. At certain museums or memorial sites, visitors are provided with VR glasses or head-mounted displays in order to supposedly reexperience the past or certain historical situations (Bunnenberg 2018, 2020). In contrast to these highly immersive VR applications, YouTube or the internet more generally provide several history-related 360° media, i.e., 360° panoramic images and 360° videos (Ardisara and Fung 2018), which users can—at a minimal immersive level—just watch on their computer or mobile device at home or in class in order to purportedly witness historical events. Although 360° media cannot be characterized as highly immersive, as they cannot be experienced through head-mounted displays (no stereoscopic perception) and enable no or reduced freedom of action (low tracking level), they fulfill other aspects that are characteristic of immersive VR (Cummings and Bailenson 2015): For example, 360° videos offer viewing and experiencing events from a first-person perspective and a wide field of view. Special VR glasses enable an ego-perspective eye movement control, whereby the entire field of view can be used. These immersive features can evoke presence and distinguish 360° videos from conventional videos (Ardisara and Fung 2018).

Due to these immersive features and the users’ feeling of presence, VR media can—in contrast to conventional history-related videos, texts, and images—intensify the users’ perceived authenticity of the represented historical contexts and their impression of reexperiencing, witnessing, and participating in historical events (Bunnenberg 2020). Thus, using VR media as educational technologies in history-learning contexts can “help learners to situate themselves in historical contexts” and to “feel more connected to the narrative” (Parong and Mayer 2021, p. 1436), whereby both affective and cognitive processing of the content may be fostered (Parong and Mayer 2021).

The immersive aspects of VR can, however, lead to challenges for learners who are asked to use VR media for learning purposes. Learners may get distracted from the learning task (Jensen and Konradsen 2018), experience motion sickness (Reiners et al. 2014), or feel unsafe due to technological aspects which block out the access of the real surroundings (Reiners et al. 2014). Emotionalization, which is a characteristic feature of representations of history-related content in (VR) media, museums, or memorial sites (Brauer 2016; Blaschitz 2016; Buchner 2019), could overwhelm learners and make it difficult for them to critically question and distance themselves from the presented content (Bunnenberg 2020). Immersive characteristics of VR, such as experiencing the environment in the first-person perspective, might increase this emotionalization and, thus, lead to a highly emotional and less cognitive processing of the history-related content (Bunnenberg 2020; Parong and Mayer 2021). It is likely that a VR experience of a historical situation from the first-person perspective and, thus, from the perspective of a historical figure, such as a gladiator, soldier, or prisoner, could lead to a high identification with these figures, an adoption of their perspectives, and, thus, strong emotions in the users. This overidentification and lack of distancing could lead to an unreflected adoption of the presented content as a valid representation of the past (Brauer and Lücke 2013; Bunnenberg 2020). Consequently, when learners are asked to learn with history-related VR media, they should be supported in critically reflecting the presented content and the characteristics of the medium.

### Strategies for processing history-related VR

In history education, the ability to critically examine and analyze history-related representations is referred to as a competence that should be acquired during historical learning (e.g., Schreiber 2008; Gautschi 2018; Waldis et al. 2012). In their model of competencies for historical learning, Gautschi et al. (2009) describe the competence of critically analyzing historical sources and, for example, the ability to characterize different types of texts and assess their epistemic value as “exploration”.

While providing important theoretical models of historical competencies, to our knowledge, research on history education has not yet empirically investigated the effectiveness of specific approaches for fostering these competencies. For this purpose, it might be fruitful and promising to consider findings from educational research on self-regulated learning (SRL) and especially on the effectiveness of SRL trainings for fostering students’ acquisition of cognitive strategies. Specifically, certain historical competencies can be linked to learning actions, which are defined as cognitive strategies in the context of SRL. SRL refers to planning and adapting one’s own learning actions in order to achieve personal goals, for which the use of learning strategies plays a crucial role (e.g., Zimmermann 2000). Learning strategies refer to behaviors of learners that are assumed to have an impact on their affective and cognitive processes during learning (e.g., Weinstein and Mayer 1986). Models on SRL often distinguish three different types of learning strategies: cognitive, metacognitive, and affective or resource management strategies. Cognitive strategies are techniques for encoding and processing information from learning materials by elaborating, organizing, and rehearsing new information (e.g., Weinstein and Mayer 1986; Pintrich et al. 1993). Metacognitive strategies are defined as techniques for planning, monitoring, and regulating one’s learning actions, such as setting a learning goal and modifying one’s learning strategies to achieve this goal (e.g., Weinstein and Mayer 1986; Pintrich et al. 1993). Affective strategies or resource management strategies concern techniques for creating an effective learning environment, such as maintaining concentration and motivation, or managing time effectively (e.g., Weinstein and Mayer 1986; Pintrich et al. 1993).

We identified interesting similarities or overlaps between certain *cognitive strategies* as defined by research on SRL and specific competencies described in the model of competencies for historical learning (Gautschi et al. 2009). For instance, the historical competence “Exploration”, which refers to the ability to critically examine, analyze, and characterize genre-specific features of history-related representations and their presented phenomena, facts, and persons (Gautschi et al. 2009), may overlap with organizational strategies (as one type of cognitive strategies), such as summarizing, structuring, or outlining, that are assumed to help learners to identify relevant facts, main ideas, and arguments in the learning material at hand (Weinstein and Mayer 1986; Wild and Schiefele 1994).

If students learn to apply those cognitive strategies—which seemingly overlap with certain competencies for historical learning—, they can use a structured approach for both reflecting their emotions as well as elaborating and contextualizing the learning content in a rather distanced and critical way. Thereby, students could develop important aspects for the historical learning process, such as being aware of the changeability of history, knowing about different perspectives, or understanding what emotions drove the people of that time (Brauer 2016; Waldis et al. 2012; Gautschi 2018). Consequently, in order to support students’ critical and reflective processing of history-related VR media, such as 360° videos, it might be beneficial to foster their acquisition of certain cognitive strategies.

### Fostering learning with history-related VR

Research on learning with history-related VR has not yet focused on investigating and promoting the methodical, critical, and reflective processing of the content, but mainly on the effectiveness of VR for interest or enjoyment (e.g., Rahimi et al. 2020; Yildirim et al. 2018). Studies that have examined the cognitive effects of using history-related VR usually focus on learning outcomes, such as remembering and reproducing information or understanding the content (e.g., Taranilla et al. 2019; Calvert and Abadia 2020; Parong and Mayer 2021). To the best of our knowledge, so far, only one empirical study has examined students’ cognitive reflection of history-related VR media: A study by Frentzel-Beyme and Krämer (2022) indicates that providing users with additional context-related information about the history-related VR application at hand has the potential to support their cognitive reflection processes (e.g., historical consciousness) while using immersive and highly emotionalizing history-related VR. Thus, more research is required that investigates powerful ways for supporting students’ methodical, critical, and reflective processing of immersive history-related VR.

One promising way may relate to fostering students’ use of cognitive strategies through a SRL training. SRL trainings have already been proven to be helpful procedures for practicing the use of cognitive strategies as well as for improving academic success and different other learning outcomes (e.g., Theobald 2021; Zheng 2016; Dignath et al. 2008). However, it has not yet been investigated whether fostering learners’ acquisition of cognitive strategies can help them to process history-related VR applications in a critical and distanced way. So far, only Dold (2020) has investigated whether fostering students’ SRL affects their deep learning at memorial sites. But Dold (2020) focused on how active and independent tours at memorial sites could be promoted and not on how students’ interaction with history-related VR could be supported.

## The present study

Against this background, the present study investigated the effects of a SRL training on student learning with immersive history-related VR media. More specifically, we focused on students’ processing of minimal-immersive 360° videos due to two reasons: Firstly, due to the pandemic, students learned in online classes at home and, thus, had no access to technology for using highly immersive VR. Secondly, beyond the feasibility of conducting a study in times of Covid-19, using VR media as they are available on the internet (e.g., 360° videos), may be a typical situation in history-education contexts that occurs around learning in class or in conjunction with homework assignments.

The primary goal of the present study was to examine whether the positive effects of SRL trainings on students’ acquisition of cognitive strategies that have been demonstrated in previous research transfer to the context of historical learning with 360° videos. Building on the argument developed above that the use of cognitive strategies may help students to process the content of immersive history-related VR in a less emotionalized and rather distanced and critical way, we additionally aimed to investigate whether a SRL training indeed affects the way how students perceive history-related 360° videos (in the following referred to as type of analysis) and how the use of cognitive strategies is related to their interpretation of the represented content and their kind of analysis.

To achieve these goals, we developed a SRL training that fostered students’ acquisition of cognitive strategies for processing history-related 360° videos. To investigate the effectiveness of this training for historical learning, we conducted a quasi-experimental study and compared two conditions: an explicit SRL training (training condition) and an implicit SRL training (control condition). While students in the training condition learned the terms and functionality of cognitive strategies and were explicitly guided to practice the use of these strategies while analyzing a history-related 360° video, students in the control condition were only implicitly guided to use cognitive strategies for their video analysis without receiving explicit instruction or information on the terms and functionality of these strategies.

In summary, we focused on the following three research questions: (RQ1) Do students who receive an explicit SRL training acquire more cognitive strategies when analyzing a 360° video than students who receive an implicit SRL training? (RQ2) Do students who receive an explicit SRL training differ from students who receive an implicit SRL training in their type of analysis of a history-related 360° video? (RQ3) How is the use of cognitive strategies connected to students’ types of analyses of a 360° video, and how does this interplay differ between students who receive an explicit SRL training and students who receive an implicit SRL training?

## Method

### Participants

164 secondary school students (52% female) from 10 schools agreed to participate with written parental consent in our study. The classes (9th to 13th grade) were randomly assigned to the training condition (five classes: *n* = 70) or the control condition (five classes: *n* = 94).

### Design and procedure

Due to the pandemic situation, the study took place in the form of an online video conference via Zoom. As shown in Table 1, the interventions in both conditions started with an introduction to historical terms. Afterwards, students in the training condition received the explicit SRL training. In accordance with findings of previous SRL research suggesting that a combined training of both metacognitive and cognitive strategies is particularly beneficial for students’ acquisition of cognitive strategies (e.g. Dignath et al. 2008; Stebner et al. 2015), our SRL training combined cognitive with metacognitive strategies. Specifically, the explicit SRL training in the training condition consisted of two parts: Students first received a strategy lecture on the terms and functionality of cognitive and metacognitive strategies, and then practiced to use these strategies for analyzing 360° videos in an explicit strategy exercise. Students in the implicit training condition participated only in an implicit strategy exercise for analyzing 360° videos without a previous strategy lecture. To keep the time of the intervention consistent in both conditions, students in the control condition received (instead of a strategy lecture) a more detailed introduction to historical terms at the beginning. Two instructors conducted the study: while one person (a doctoral student in history education) conducted the history-related introduction and the exercise, the other person (a research assistant in educational psychology) conducted the strategy lecture and administered the Strategy Use Test during the online conference (see Table 1). The instructors and their respective responsibilities did not change throughout the whole study.

**Table 1:** Procedure in both conditions

	Training condition (explicit SRL training)	Control condition (implicit SRL training)	Use of 360° media
*Prior to the online conference*	Strategy Use Test & Strategy Knowledge Test (not focused on in this paper)		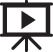
*During the online conference (90* *min.)*	Introduction	Introduction	–
	Strategy lecture		
	Exercise (explicit strategy practice)	Exercise (implicit strategy practice)	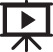
	Strategy Use Test		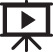
*After the online conference*	Strategy Knowledge Test (not focused on in this paper)		–

During the introduction, the instructor discussed the differences between the terms of *history* and *past *with students in both conditions and introduced 360° videos as a special form of representation.

During the strategy lecture, students in the training condition received an interactive ten-minute lecture on the definition of learning strategies and the functionality and relevance of cognitive and metacognitive strategies for historical learning. Students in the implicit training condition did not receive this lecture, but received implicit guidance on the use of learning strategies during the exercise (see the following paragraph).

During the exercise, students in the training condition practiced to analyze 360° videos and were explicitly guided to use learning strategies. Students in the control condition practiced to analyze 360° videos, but were only indirectly guided to use learning strategies. That is, students in the control condition received guidance on the use of metacognitive and cognitive strategies, but the instructor did not explicitly use the corresponding terms of the strategies or explain their functionality. For instance, the instructor in the control condition implicitly guided students to use the metacognitive and cognitive strategies of goal setting and summarizing the content by asking the following question: “Can somebody summarize what this video is about?” Instead, in the explicit training condition, the instructor formulated the following questions: (1) “In order to apply all the strategies we have learned, we must first set ourselves a goal, that is: examining the video to see what image of history it conveyed. Why is this strategy so important?” and (2) “As a next step, we want to use the strategy ‘summarizing the content’. Can somebody remember what this strategy is about and why it is relevant for our goal we just set?” Thus, in both conditions, the exercise asked students to practice the use of the strategies, but while students in the training condition received explicit guidance on the use of these strategies, students in the control condition received only implicit guidance during the exercise.

Prior to the online conference, we administered a pretest assessing students’ *use* of cognitive strategies and their *knowledge* about cognitive strategies in both conditions. At the end of the two interventions, students in both conditions were asked to work on an immediate posttest that measured their *use* of cognitive strategies. After the online conference, a second posttest assessed students’ *knowledge* about cognitive strategies in both conditions. The two Strategy *Use* Tests (i.e., pretest and posttest) required students in both conditions to watch a history-related 360° video and to analyze the video with respect to the conveyed view of history. The two Strategy *Knowledge* Tests asked students to explain how to analyze a 360° video in general. However, the analyses presented here only focus on students’ performance on the immediate Strategy Use Test after the intervention.

### Learning materials

In both conditions, we used a digital poster of a streaming series for the introduction and a 360° video for the exercise. In the explicit training condition, we additionally provided students with a strategy handout. All learning materials were included in a PowerPoint presentation which the instructors used during the interventions.

The poster aimed to motivate students to participate in the online conference and to exemplify the differences between a historical image and the actual past. That is, the poster was about a series that refers to historical events of the Viking Age, but it contains fictional and unproven events. The instructor asked students to discuss (in the whole class) what kind of image about the time of the Viking Age the poster conveyed and to share their impressions about the authenticity of the representation.

**Fig. 1:**
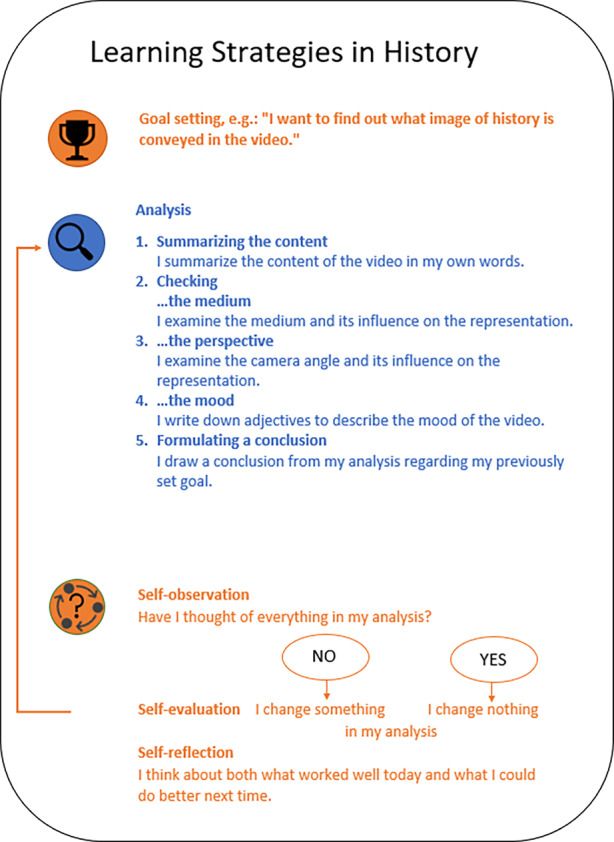
Handout showing the learning strategies focused on in the explicit training condition

The strategy handout (see Fig. 1), which builds on the experimentation strategy developed by Stebner et al. (2015), included an overview of the cognitive and metacognitive strategies that students in the explicit training condition should acquire for analyzing a history-related 360° video. The instructor employed the handout for illustrating the strategic procedure of the analysis of a history-related 360° video during the strategy lecture in the explicit training condition with the goal to prepare students for the following practical exercise. For this purpose, the instructor first showed a simplified version of the handout that introduced learning with metacognitive and cognitive strategies in general. Afterwards, the students received a specific version of the handout showing the cognitive and metacognitive strategies relevant for the analysis of history-related 360° videos (see Fig. 1). Based on the definition of historical learning (Gautschi et al. 2009), elements of film analysis (Teuscher 2006; Buchner 2015), and the definitions of certain cognitive and metacognitive strategies in SRL research (e.g., Wild and Schiefele 1994; Friedrich and Mandl 2006), the handout and, thus, the explicit SRL training focused on five cognitive strategies and four metacognitive strategies (see Fig. 1). During the exercise in the explicit training condition, students practiced to apply these strategies gradually with the help of the instructor.

During the exercise in both conditions, we used the video “The Beginning of the Wall” which is available on YouTube and is about the early days of the German Democratic Republic and German citizens in East Berlin who were prevented from leaving the communist state. It should be mentioned that the video actually consists of programmed 360° panoramic images arranged in series and underlaid with audio content. However, it contains characteristic features of immersive history-related videos, such as a view from the first-person perspective, the ability to rotate the viewing by mouse click, and an emotionalizing representation of the content. Due to the pandemic conditions, students opened the video on their desktop and watched it without using cardboards. The instructor asked students to analyze the video in a whole-class discussion, and guided students—either explicitly in the training condition or implicitly in the control condition—to use the metacognitive and cognitive strategies.

### Measures

To investigate our research questions, we analyzed the student answers in the Strategy Use Posttest. The test, which is based on the multi-strategy test developed by Stebner et al. (2015), asked students to first watch the 360° video “What did they [members of the German State Security Service] want in Berlin?!” (available on YouTube) and then to analyze its presentation of the past. We coded the student answers in two rounds of analysis. We first developed, in the context of a previously conducted implementation check (see Yek et al. 2022), categories for measuring students’ use of cognitive strategies for analyzing history-related 360° videos. For the analyses present here, we then additionally developed categories for measuring students’ types of analyses.

For assessing students’ *use of cognitive strategies*, we coded students’ answers through a top-down process and used—based on the contents of our SRL training—a system of five categories, namely one category for each cognitive strategy. Table 2 shows the respective codebook. We coded the answers in a binary way and, thus, only coded whether one of the strategies occurred (1) or not (0) within a student answer. Two independent raters coded 30% of the posttest dataset and reached, on average, a satisfying agreement (κ = 99.88).

**Table 2:** Category system for analyzing the use of cognitive strategies

Strategy	Code definition	Examples of student answers
Summarizing the content	Give a brief introductory presentation of the video and its content (e.g., title, type of medium, topic)	“In this 360° video, you take the role of a person who goes to prison in the course of the video”. “The video ‘What did they want in Berlin?!’ is about a man who had to go to prison.”
Checking the medium	Describe or analyze the medium and its influence on the representation	“The whole situation is presented as a 360° video, which means that the viewer can look around and has an all-round view during the event.” “This is a 360° video.”
Checking the perspective	Describe the kind of perspective (e.g., first-person perspective) or analyze what influence the camera setting/first-person perspective might have on the interpretation	“The video is from the point of view of a prisoner in the DGR.” “This camera perspective gives you a different feeling of being there: You are a prisoner yourself and see everything from his point of view. You can also empathize better with the situation.”
Checking the mood	Describe or analyze the atmosphere created in the video	“The atmosphere is very tense […].” “The dark sounds and the environment seem gloomy and scary.”
Formulating conclusions	Draw at least one conclusion with regard to the task	“The Stasi does not seem to grant any human rights to the prisoners and the conditions in this prison also seem to be bad.” “Overall, I draw from this video experience that it was very strict and guarded in Berlin back then.”

For assessing students’ *types of analyses,* we generated (through a bottom-up process) five categories that build on the actual data. Table 3 shows our codebook. We again coded the answers in a binary way. Two raters coded 100% of the dataset and reached, prior to discussion, satisfying agreements with interrater reliability values for the five codes ranging between κ = 0.75 and κ = 1.0. However, prior to analysis, all disagreements were resolved by discussion.

**Table 3:** Category system for analyzing the types of analysis

Code (type of analysis)	Code definition	Examples of student answers
Superficial analysis	For most parts, students describe or summarize the video superficially and on the content level only (lack of meta-level); they do not address the different modes of action of the presentation	“In the 360° video there are different places like an office where you are threatened and questioned. Then there is also a room where you are photographed. There is also a room where you are detained. Many people had to undress.”
Emotionally-immersed focus	Students address the mode of action of the representation, but strongly relate to emotional aspects and/or include themselves as part of the environment (as a form of immersion) without explicitly addressing their perspective and its function	“I perceived the 360-degree video as very intimidating and gloomy. The interaction towards each other there is very aggressive. The voices are loud and threatening. Everything seems very cold and monotonous. The interaction with the people is without emotion, and you have the feeling that emotions are suppressed altogether.”
Reflective formulation	Students address different aspects of the video. They justify and explain their interpretations. They express emotional aspects of the presentation or their own feelings in a conscious and reflective way. Their analysis does not solely rely on an immersive experience of the representation	“The video is about a prisoner who is imprisoned in a Stasi prison. […] Since you have the feeling of being the prisoner yourself, you only get the impression of how the prisoner perceives the situation, because the other side remains hidden. In general, the tone of the guards is very commanding and aggressive, which reinforces the overall impression. Overall it feels very oppressive which is brought about by the white light. So the image presented of the Stasi is that of a brutal organization that sees itself in the full right and does not shy away from threats, while at the same time trying to appear to the outside world as if it was only interested in the welfare of the population.”
Acceptance of represented past	Students make statements that indicate an unreflected adoption of the video content as representation of the actual past	“The video shows how people in the GDR were treated who rebelled against the government or did not comply. […]”
Trivial or imprecise answers	Answers that cannot be assigned to codes because they consist of only a few unrelated keywords or a short sentence	“It seems aggressive and rude”

It should be noted that the two codes *superficial analysis* and *reflective formulation *are mutually exclusive, because, according to research on history learning (Gautschi et al. 2009; Mierwald and Brauch 2015; Lücke 2012) it is not sufficient for a reflective analysis to exclusively and superficially describe the video content. Instead, a reflective analysis by its definition also includes some kind of a structured, argumentative approach (Mierwald and Brauch 2015) which enables one to recognize a coherent, reasoned and logical structured analysis (Lücke 2012; Gautschi et al. 2009). As a reflective formulation includes, for example, statements about emotional aspects or the immersive experience (see Table 3), the two codes *reflective formulation* and *emotionally-immersed focus* are also mutually exclusive.

## Results

### Research question 1: use of cognitive strategies

With respect to our first research question, we conducted two analyses. We firstly conducted a Mann-Whitney‑U test and examined whether students of the training condition used more cognitive strategies for analyzing a history-related 360° video than students of the control condition. We secondly conducted a Chi-square test of independence and examined whether students of the training condition used each of the five cognitive strategies more often than students of the control condition. With regard to the total number of applied cognitive strategies (max. 5), the Mann-Whitney‑U test revealed a significant difference (*U* = 2092.00, *Z* = −2.70, *r* = 0.22 *p* = 0.007) between the students of the training condition (*n* = 66, *M* = 1.62, *SD* = 1.21) and their counterparts of the control condition (*n* = 83, *M* = 1.07, *SD* = 0.85). Thus, students who received the explicit SRL training used significantly more strategies for analyzing the history-related 360° video than students who received an implicit SRL training. With regard to each cognitive strategy (see Table 4 for descriptive statistics), the Chi-square test revealed significant differences (favoring the training condition) in using the strategies summarizing the content (X^2^ (1) = 5.97, *p* = 0.015, φ = −0.200), checking the medium (X^2^ (1) = 11.26, *p* = 0.001, φ = −0.275), and checking the perspective (X^2^ (1) = 4.15, *p* = 0.042, φ = −0.167). We found no significant differences between the two conditions in using the strategies checking the mood (X^2^ (1) = 1.05, *p* = 0.305, φ = −0.084) and formulating conclusions (X^2^ (1) = 0.24, *p* = 0.624, φ = −0.040). Hence, students of the training condition used three out of five cognitive strategies significantly more often than students of the control condition.

**Table 4:** Descriptive statistics for cognitive strategies used by students

Cognitive Strategy	Training condition (*n* = 66)	Control condition (*n* = 83)
	*M* (*SD*)	
Summarizing the content	0.23 (0.42)	0.08 (0.28)
Checking the medium	0.21 (0.41)	0.04 (0.19)
Checking the perspective	0.18 (0.39)	0.07 (0.26)
Checking the mood	0.39 (0.49)	0.31 (0.48)
Formulating conclusions	0.61 (0.49)	0.57 (0.50)

### Research question 2: types of video analyses

To investigate our second research question, we conducted a Chi-square test of independence and examined whether the types of analyses differed between students of the training and control condition. Table 5 shows the descriptive statistics and the results of the Chi-square test.

**Table 5:** Descriptive statistics for types of analyses conducted by students

Type of analysis	Training condition (*n* = 66)	Control condition (*n* = 83)	Chi-square test statistics (*df* = 1, *N* = 149)
	Absolute and relative frequencies		
Trivial or imprecise answers	3 (4.5%)	17 (20.5%)	X^2^ = 8.04, *p* = 0.005, φ = 0.23
Superficial analysis	27 (40.9%)	23 (27.7%)	X^2^ = 2.87, *p* = 0.090, φ = −0.14
Reflective formulation	18 (27.3%)	14 (16.9%)	X^2^ = 2.36, *p* = 0.124, φ = −0.13
Acceptance of represented past	19 (28.8%)	15 (18.1%)	X^2^ = 2.40, *p* = 0.122, φ = −0.13
Emotionally-immersed focus	39 (59.1%)	45 (54.2%)	X^2^ = 0.36, *p* = 0.551, φ = −0.05

As shown in Table 5, students of the training condition only differed significantly (with a small effect) from students of the control condition with regard to trivial or imprecise answers (see last row in Table 5). Specifically, students with explicit SRL training gave significantly less trivial or imprecise answers in their video analyses than students with implicit SRL training. Although not statistically significant, the descriptive statistics and especially the relative frequencies suggest that students of the training condition tended to make more superficial analyses, reflective statements, and interpretations of the video content as accepted representation of the past than students of the control condition. With regard to analyses with emotionally-immersed focus, students did neither significantly nor descriptively (i.e., students of the training condition conducted only marginally less analyses of this type than students of the control condition) differ between the two conditions.

### Research question 3: types of analyses in relation to the use of cognitive strategies

**Fig. 2:**
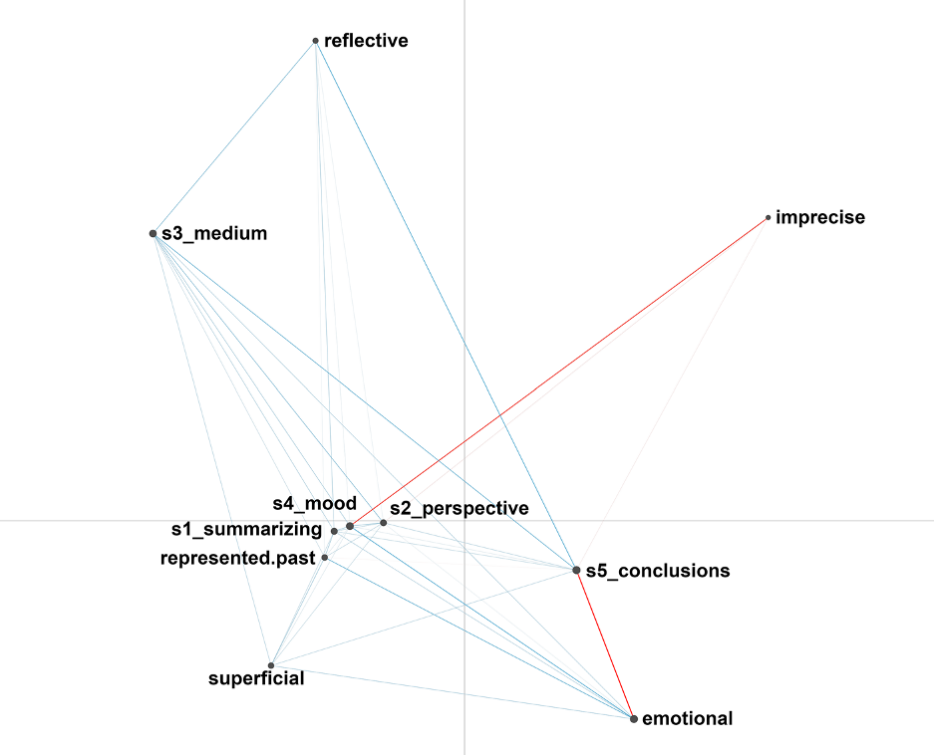
ENA difference graph for the coded video analyses of students with explicit SRL training (*blue*) and students with implicit SRL training (*red*)

To investigate our third research question, we conducted Epistemic Network Analysis (ENA) which allows to identify and measure the co-occurence and, thus, the connections between elements in coded data (Shaffer et al. 2016). ENA models the weighted structure of these connections and illustrates the respective structure in dynamic network models (Shaffer and Ruis 2017; Shaffer et al. 2016). Hence, by conducting an ENA, we were able to explore the interplay between students’ use of the five cognitive strategies and their types of video analyses and to compare this interplay between the training condition and the control condition. For this purpose, we constructed mean epistemic networks for each condition and then subtracted the networks in order to make the differences between the two conditions salient. In the resulting difference graph (see Fig. 2), the darker and thicker lines illustrate larger differences in the strength of connections, and the color of the lines illustrates in which network the connections are stronger (Shaffer et al. 2016).

As Fig. 2 shows, students with implicit SRL training (red) draw slightly more often than students with explicit SRL training conclusions from an emotionally-immersed point of view. They additionally focus more strongly than students with explicit SRL training on describing the mood of the video in an imprecise (or trivial) way. On the contrary, students with explicit SRL training (blue) draw their conclusions slightly more often than students with implicit training in a reflective way, and relate their conclusions more often to aspects of the medium. They further take slightly more often than students with implicit training an emotionally-immersed perspective in combination with both the use of the strategy checking the “mood” and an interpretation of the video as accepted representation of the past.

A t-test showed that students with explicit SRL training (*M* = −0.15, *n* = 66) significantly differed from students with implicit SRL training (*M* = 0.12, *n* = 83) on the first (x) dimension with a moderate effect (*t* *(145.23)* = −4.51, *p* < 0.01, *d* = 0.73). This significant difference on the first (x) dimension suggests that students of the training condition have their strongest connections in the left part of the space and students of the control condition in the right part of the space, consequently (and as illustrated by the difference graph), while students with explicit SRL training made more connections between the different cognitive strategies and reflective but also superficial analyses and interpretations of the video as accepted representation of the past, students with implicit SRL training made more imprecise analyses and emotionally-immersed conclusions.

The answers depicted in Table 6 from two students of the explicit training condition exemplify the connections (as illustrated by the previous ENA) between the use of different cognitive strategies, a reflective analysis, and the interpretation of the video content as accepted representation of the past.

**Table 6:** Example video analyses conducted by students of the explicit training condition

Student	Video analysis	Cognitive strategies	Type of analysis
#1	“With this video, I want to find out what it was like in Berlin at that time with the Stasi. At the beginning, I am in a prison corridor. Three policemen stare at me and are very rude. You can see this kind of manners throughout the video. Other places include an office where I am questioned and examined. After that a picture is taken of me and at the end, I end up in a prison cell. The whole situation is presented as a 360° video, which means that the viewer can look around during the whole event and has a panoramic view. This gives you the opportunity to take a closer look at everything and not just experience it from one perspective. Due to this camera perspective, one directly has a different feeling of witnessing. You are a prisoner yourself and see everything from your point of view, and you also feel more involved in the situation. If this had only been a picture, one could not have understood so well how the prisoner must have felt or how rude the police officers were.. Colour-wise it is very faded, and a negative mood builds up as a result. The rooms and corridors are empty and guarded. That’s why the whole video makes you feel very uncomfortable and tense. Because you are also shouted at and insulted, it is a very authoritative atmosphere. Overall, I learn from this video experience that it was very strict and guarded in Berlin at that time. No one had privacy and the police, and the Stasi had a lot of power.”	Summarizing the content, checking the perspective, checking the medium, checking the mood, formulating conclusions	Reflective formulation, acceptance of represented past
#2	“This video is about a man who was recorded in a prison. He remains silent and does not say anything. The State Security Service demands him to undress. It is filmed from the man’s perspective, so he is in the middle of the action. The tone of the officers is loud and strict. Therefore, the atmosphere is very tense and not really relaxing. The whole situation seems very dull and emotionless. In the end, you can say that this used to be a place where you wouldn’t like to be.”	Checking the perspective, checking the mood, formulating conclusions	Reflective formulation, acceptance of represented past

Student 1 started his or her analysis with a goal formulation and an orientation in the virtual space, while student 2 gave an introductory summary about the video content (“This video is about …”). Student 2 then addressed the perspective and mood in the video, and reflected on the respective impact of the perspective and mood on the viewer’s perception of the video and the viewer’s own emotions. Student 1 proceeds in a similar way, but in more detail and without an introductory summary. Student 1 conducts a rather descriptive and emotionally distanced analysis, but writes the analysis from a first-person perspective, which could suggest an immersion in the video. The reflective features of the analysis and its connection to the strategies *checking the medium, checking the perspective, checking the mood* and *formulating conclusions *is particularly apparent in the middle sections of the analysis given by student 1. Student 1 also addressed various aspects of the presentation, formulated and justified his or her own interpretations, and even considered the impact of the medium on his or her own interpretation. Thus, this analysis combined mainly reflective statements with the use of different cognitive strategies. Nevertheless, at the beginning in the answer of student 1 and especially at the end of the analyses of student 1 and student 2, both formulated statements that indicate an *acceptance of the represented past*. The formulated goal of finding out what it was like back then (student 1) is a first indication for this acceptance. A reflective realization that it is impossible to find out how it really was back then through the video is also missing after or at the end of the analysis given by students 1, as he or she still concluded that “it was very strict and guarded in Berlin at that time” and “no one had privacy […]”. Student 2 initially seemed to interpret the content in a critical-reflective and distanced way (e.g., “seems to be …”). However, at the end of the analysis he or she makes a rigid conclusion without referring to the content of the video anymore, but to the perceived actual past. Hence, although the two students conducted a mostly reflective and sophisticated analysis of the video, they still ended up with an unreflective perception of the represented content.

Table 7 shows exemplary answers from four students of the implicit training condition. The analyses conducted by student 3 and student 4 exemplify the connection of using the cognitive strategy *formulating conclusions* and an emotionally-immersed type of analysis. Specifically, the two students give a fairly brief analysis that is focused on emotional aspects of the representation. In addition, both answers are linked to conclusions about the conveyed image of the past. A reflective as well as distanced analysis of certain features of the representation is missing.

**Table 7:** Example video analyses conducted by students of the implicit training condition

Student	Video analysis	Cognitive strategies	Type of analysis
#3	“A very gloomy picture is conveyed. It seems as if the person who was talked to was brought to prison. This person was threatened and treated very unkindly. They were told that they couldn’t harm the regime.”	Formulating conclusions	Emotionally-immersed focus
#4	“A very paranoid picture is presented. No matter what you did, there were probably ulterior motives: For example, only because I was in the capital I was supposed to have planned something. The rooms that were shown looked very cold and the people were unfriendly. They were very rough with you.”	Formulating conclusions	Emotionally-immersed focus
#5	“Everything is rough and desolate.”	Checking the mood	Trivial or imprecise answer
#6	“They wanted to scare you.”	–	Trivial or imprecise answer

The answers from students 5 and 6 exemplify an imprecise or trivial type of analysis, as both answers consist of a keyword-like sentence that, although seemingly related to the content of the video, is phrased incoherently. Student 5 describes the mood in the video very briefly, and student 6 is probably referring to the characters shown in the video. However, both statements are insufficient for being coded as a superficial, emotionally-immersed, or reflective type of analysis.

## Discussion

Due to the increased use of VR in educational contexts and the challenges that go along with the processing of those immersive media, students need appropriate skills in order to process the represented contents in a critical and reflective way, especially in the context of history education (e.g., Bunnenberg 2020). Building on history-education research (e.g., Gautschi et al. 2009) and research related to self-regulated learning (SRL) (e.g., Dignath et al. 2008; Schuster et al. 2020; Theobald 2021), the present study investigated the effectiveness of an explicit SRL training on students’ processing of history-related 360° videos. We compared the explicit training with an implicit SRL training and examined students’ use of cognitive strategies while analyzing minimal-immersive history-related 360° videos (RQ1), and the way how students processed and perceived the emotionalizing representation of the content in the 360° video (defined as types of analysis) (RQ2). We additionally explored and compared the interplay between the applied cognitive strategies and the types of analyses between the two conditions (RQ3). Thereby, we aimed to explore both, whether the beneficial effects of SRL trainings on students’ use of cognitive strategies demonstrated by previous research (e.g., Stebner et al. 2015; Dignath et al. 2008) transfer to historical learning with immersive VR, and whether the use of cognitive strategies actually leads to a more critical-reflective and less emotionally-immersed perception of the content represented in the videos. In the following sections, we summarize and discuss the findings of our study. In the subsequent chapters, we then describe the limitations of our study and finally conclude with discussing the relevance of our findings in the context of digital learning.

Our findings indicate that the explicit SRL training affected students’ use of cognitive strategies when analyzing a history-related 360° video (RQ1). This is suggested by both a higher total number of applied strategies and the higher use of certain strategies: Students in the explicit training condition used the strategies *summarizing the content, checking the medium,* and *checking the perspective* more often than their counterparts in the implicit training condition. These findings are in line with previous research demonstrating beneficial effects of SRL trainings on students’ strategy use (for a review, see Dignath et al. 2008), and suggest that these beneficial effects of SRL trainings can be transferred from the mathematics and reading/writing contexts of previous research (see Dignath et al. 2008) to history-related learning contexts. As the cognitive strategies that we aimed to foster by our SRL training overlap and build on abilities that are referred to as competencies for historical learning (e.g., Gautschi et al. 2009), the results of the present study also demonstrate that our SRL training has the potential to promote the acquisition of specific historical competencies. Specifically, our SRL training promoted the acquisition of the exploration competence—as one specific competence for historical learning—which refers to the ability to analyze and characterize genre-specific features of history-related representations and their presented phenomena, facts, and persons (Gautschi et al. 2009; Waldis et al. 2012). In light of the short duration of the explicit SRL training (10-minute strategy lecture followed by a 30-minute exercise), the positive effect of our SRL training on students’ use of cognitive strategies for analyzing history-related 360° videos is particularly remarkable. Further research is required to investigate whether an extended training time could lead to even higher effects on students’ use of cognitive strategies (see Dignath et al. 2008) for analyzing history-related VR media. The positive effects of our explicit SRL training are moreover particularly remarkable as the present study did not use a “lousy control condition” (see Schwonke et al. 2009). More specifically, students in both conditions received training (either explicitly or implicitly) on the use of cognitive strategies for analyzing history-related 360° videos. Thus, the demonstrated effects of our explicit SRL training cannot be reduced to being an artefact of a weak control condition in which students had no chance to learn. However, the fact that students of the control condition had also received implicit training on the use of cognitive strategies for analyzing 360° videos could be one reason for our further results showing no significant differences between the two conditions with regard to the use of the strategies *checking the mood* and *formulating conclusions.* Due to the implicit training, students of the control condition might have indirectly learned to use certain strategies, such as checking the mood or formulating conclusions, while analyzing a history-related 360° video. Another reason for the non-significant difference in using the strategy *checking the mood* could relate to the immersive and highly emotionalizing character of the video, such that students of the control condition intuitively described the atmosphere of the video in their analysis. The non-significant difference in using the strategy *formulating conclusions* may be due to the task instructions. Specifically, students were asked to analyze the image of the past that is conveyed in the video. By referring to this task, they automatically—as defined in our codebook (see Table 2)—formulated a conclusion.

The findings of the present study additionally suggest that the explicit SRL training and, thus, the use of cognitive strategies probably helped students to conduct their video analysis in a more structured way than with an implicit training, as they gave significantly less imprecise or trivial answers (RQ2), and at the same time connected more of the cognitive strategies with their different types of analyses than students of the control condition (RQ3). The explicit SRL training seemingly also helped students to draw more reflective conclusions. Although they did not conduct significantly (but descriptively) more reflective analyses than students of the control condition (RQ2), our ENA revealed that students with explicit SRL training used slightly more often than students with implicit SRL training the cognitive strategy* formulating conclusions* for a reflective type of analysis (RQ3). Specifically, while 23% of the training condition (i.e., 15 out of 66 students) drew reflective conclusions, only 12% of the control condition (i.e., 10 out of 83 students) did the same. However, our analyses with regard to RQ2 and RQ3 suggest rather small differences in the types of analyses (in relation to students’ use of cognitive strategies) between the explicit and the implicit training condition. Moreover, as particularly shown by our qualitative analyses of exemplary video analyses, students of the training condition still included statements in their analyses indicating that they accepted the represented past without questioning it. In addition, similar to students of the implicit training condition, they often focused on emotionally-immersed perceptions of the video (see Table 5). One possible reason for this finding might relate to the short duration of the training. Specifically, it is likely that the explicit training helped students to develop an awareness of the differences between the terms *history* and *image of the past*, and also to get a first impression of how to critically deal with the respective representations. However, they probably would have needed more time to deeply internalize and translate this knowledge into practice. The rather high number of emotionally-immersed types of analyses further indicates that students may need—in addition to guidance on the use of cognitive strategies—explicit support in reflecting on their own emotions and the emotions conveyed in immersive history-related VR in order to process the represented content in a critical, reflective, and distanced way. Thus, on the one hand, our findings with respect to RQ2 and RQ3 suggest that the acquisition and application of cognitive strategies can be a starting point for helping students to critically process immersive history-related media (as they conducted more structured analyses and drew slightly more reflective conclusions) and, thereby to encounter the potential dangers that arise from both immersive features of VR media and emotionalizing representations of history-related content (e.g., overidentification, highly emotional processing, getting overwhelmed, etc.: see Bunnenberg 2018, 2020; Brauer and Lücke 2013; Brauer 2016). On the other hand, given the small differences between the two conditions, the rather unreflected acceptance of the represented past, and the high number of emotionally-immersed types of analyses, the processing of the videos by students of the training condition cannot be described as sufficiently critical and reflected. These findings are in line with research on history education emphasizing that historical learning and the acquisition of corresponding competencies is a complex and demanding endeavor (e.g., Gautschi et al. 2009; van Drie and van Boxtel 2008; Mierwald and Brauch 2015).

## Limitations

As mentioned in the previous discussion of our findings, a limitation of the present study may relate to the rather short duration of the implemented (explicit) SRL training. Due to the pandemic restrictions, we conducted our study and, thus, the SRL training in an online format. Thereby, we had to conduct our study in the timeframe of a regular history lesson and, thus, had to limit the online conference to 90 minutes and the implemented training to a maximum of 60 minutes . However, as the effectiveness of SRL trainings depends partly on the length of time (e.g., Dignath et al. 2008), future studies should investigate whether the positive effects of our training can be increased by extending the time of the training. Due to the format of our online study, we, moreover, were only able to randomly assign the students to our two conditions on a class level. Thus, a further limitation of our study may relate to the nested character of the data. However, results of a previously conducted implementation check revealed no significant pretest differences between the two conditions with regard to students’ use of and knowledge about cognitive strategies (see Yek et al. 2022).

## Conclusion

VR media are not only increasingly used in history-learning contexts (Bunnenberg 2020), but also politically desired and applauded (Schulministerium NRW 2021). Given this situation, and in light of the topic of this special issue, we would like to conclude that it is indispensable to investigate the conditions and prerequisites for employing VR in a beneficial and meaningful way for learning. We think it is of great relevance to link the implementation of highly praised digital technologies, such as VR media in history-learning contexts, with well-established knowledge from educational research on methods and principles of instruction in order to support a differentiated, well-grounded use of these educational technologies. The present study focused on one aspect of such a research agenda: we linked the use of history-related minimal-immersive 360° videos, which are increasingly available on the internet and can be watched at home on computers or mobile devices, with knowledge from empirical research on SRL. Specifically, we examined the effects of a SRL training on students’ use of cognitive strategies for critically analyzing history-related 360° videos. Our study, thereby, tried to address both goals of history education (e.g., fostering students’ ability to critically examine history-related representations) and potential dangers of using immersive VR media (e.g., lack of distancing from presented content). Based on the findings of the present study, we have a first indication that a SRL training can be promising in order to support students’ processing of minimal-immersive history-related VR media.

However, our study is only a first step in bridging between the possibly exaggerated promises of using VR media and well-established knowledge from educational research. Further research is needed to investigate whether the effects demonstrated in our study can be replicated and possibly increased by extending the time of the SRL training, or by focusing on students’ critical reflection on their emotions and the emotionalization of the immersive representation. In future studies it would also be interesting to investigate the impact of further factors, such as the degree of immersion of the media or students’ attitudes towards immersive media, on the effects of a SRL training on students’ processing of history-related VR media.

In summary, the findings of our study contribute to three different areas: (1) Concerning learning with digital technologies, our study shows that the use of immersive media for learning can be supported by fostering students’ acquisition of cognitive strategies. (2) In the area of educational research, our study extends research on SRL by demonstrating that the beneficial effects of SRL trainings can be transferred to history-related learning contexts. (3) Our study also contributes to empirical research on history education by demonstrating that a SRL training could be a promising approach for fostering competence-oriented historical learning. Hence, teachers could use, adapt, and extend our SRL training such that students could use a strategic approach for analyzing different emotionalizing representations in history class.

## References

[CR1] Ardisara A, Fung FM (2018). Integrating 360° videos in an undergraduate chemistry laboratory course. Journal of Chemical Education.

[CR2] Black, E. R. (2017). *Learning then and there: An Exploration virtual reality in K‑12 history education*. Texas [dissertation].

[CR3] Blaschitz E (2016). Mediale Zeugenschaft und Authentizität [Media testimony and authenticity. Contemporary historical mediation work in augmented everyday space]. Zeitgeschichtliche Vermittlungsarbeit im augmentierten Alltagsraum. Hamburger Journal für Kulturanthropologie (HJK).

[CR4] Brauer J, Eggert KH (2016). ‘Heiße Geschichte‘? Emotionen und historisches Lernen in Museen und Gedenkstätten [Emotions and historical learning in museums and memorials]. Edition Historische Kulturwissenschaften.

[CR5] Brauer J, Lücke M, Brauer J, Lücke M (2013). Emotionen, Geschichte und historisches Lernen. Einführende Überlegungen. [Emotions, history and historical learning. Introductory Considerations. Geschichtsdidaktische und geschichtskulturelle Perspektiven.

[CR6] Buchner J (2015). Buchners Geschichte Oberstufe. [Buchner’s history senior classes].

[CR7] Buchner J, Schön L, Lesk S (2019). Workshop: Geschichte erleben mit Augmented und Virtual Reality [Workshop: Experience history with augmented and virtual reality]. „Retten uns dir Phänomene?“ Lehren und Lernen im Zeitalter der Digitalisierung.

[CR8] Bunnenberg, C. (2018, February 1). Virtual time travels? Public history and virtual reality. *Public history weekly, 6*(3). https://public-history-weekly.degruyter.com/6-2018-3/public-history-and-virtual-reality/. Accessed 25 July 2022.

[CR9] Bunnenberg, C. (2020). Mittendrin im historischen Geschehen? Immersive digitale Medien (Augmented Reality, Virtual Reality, 360°-Film) in der Geschichtskultur und Perspektiven für den Geschichtsunterricht. [Right in the middle of historical events? Immersive digital media (augmented reality, virtual reality, 360° film) in historical culture and perspectives for history education]. *geschichte für heute, 13*(4), 45–59.

[CR10] Calvert J, Abadia R (2020). Impact of immersing university and high school students in educational linear narratives using virtual reality technology. Computers and Education.

[CR11] Cummings J, Bailenson J (2015). How immersive is enough? A meta-analysis of the effect of Immersive technology on user presence. Media Psychology.

[CR12] Di Natale C, Repetto C, Riva G, Villani D (2020). Immersive virtual reality in K-12 and higher education: a 10-year systematic review of empirical research. British Journal of Educational Technology.

[CR13] Dignath C, Büttner G, Langfeldt HP (2008). Components of fostering self-regulated learning among students. A meta-analysis on self-regulation training programmes. Educational Research Review.

[CR14] Dold C (2020). Außerschulische Lernorte neu entdeckt. Wie selbstreguliertes Lernen in Gedenkstätten tiefgreifende Lernprozesse fördert. [Extracurricular places of learning rediscovered. How self-regulated learning in memorials promotes profound learning processes].

[CR38] van Drie J, van Boxtel C (2008). Historical Reasoning: Towards a&nbsp;framework for analyzing students’ reasoning about the past. Educational Psychology Review.

[CR15] Frentzel-Beyme L, Krämer N (2022). Ready for a&nbsp;time journey? Potentials and impact of historical VR applications on history education and morality.

[CR16] Gautschi P, Werner D, Laumer A, Wein M (2018). Videotaped eyewitness interviews with victims of national socialism for use in schools. Interactions: explorations of good practice in educational work with video testimonies of victims of national socialism.

[CR17] Gautschi P, Hodel J, Utz H (2009). *Kompetenzmodell für „Historisches Lernen“ – eine Orientierungshilfe für Lehrerinnen und Lehrer *[Model of competencies for historical learning—Orientation for teachers.

[CR18] Hartmann C, Bannert M (2022). Lernen in virtuellen Räumen. Konzeptuelle Grundlagen und Implikationen für künftige Forschung [Learning in virtual spaces. Conceptual foundations and implications for future research]. MedienPädagogik: Zeitschrift für Theorie Und Praxis Der Medienbildung.

[CR19] Jensen L, Konradsen F (2018). A review of the use of virtual reality head-mounted displays in education and training. Education and Information Technologies.

[CR20] Liu D, Bhagat KK, Gao Y, Chang T-W, Huang R, Liu D, Dede C, Huang R, Richards J (2017). The potentials and trends of virtal reality in education. Virtual augmented, and mixed realities in education.

[CR21] Lücke M, Barricelli M, Lücke M (2012). Multiperspektivität, Kontroversität, Pluralität [Multiperspectivity, controversy, plurality. Handbuch Praxis des Geschichtsunterrichts.

[CR22] Friedrich HF, Mandl H, Mandl H, Friedrich H (2006). Lernstrategien: Zur Strukturierung des Forschungsfeldes [Learning strategies: To structure the research field. Handbuch Lernstrategien.

[CR23] Mierwald, M., Brauch, N. (2015). „Ich denke, dass Anne Franks Tagebücher eigentlich eine sehr gute Quelle sind, da …“ – Zur Konzeptionalisierung und Förderung des historischen Argumentierens im Fach Geschichte [“I think that Anne Frank’s diaries are actually a very good source because …”—To conceptualize and promote historical argumentation in history]. In A. Budke, M. Kuckuck, M. Meyer, F. Schäbitz, K. Schlüter & G. Weiss (Eds.), *Fachlich argumentieren lernen. Didaktische Forschungen zur Argumentation in den Unterrichtsfächern* (pp. 215–229). Münster: Waxmann.

[CR24] Parong J, Mayer R (2021). Learning about history in immersive virtual reality: does immersion facilitate learning?. Education Tech Research Dev.

[CR25] Pintrich PR, Smith DAF, Garcia T, McKeachie WJ (1993). Reliability and predictive validity of the motivated strategies for learning questionnaire (Mslq). Educational an Psychological Measurement.

[CR26] Rahimi FB, Boyd JE, Levy RM, Eiserman JR (2020). New media and space: an empirical study of learning and enjoyment through museum hybrid space. IEEE Trans Vis Comput Graph.

[CR27] Reiners T, Wood LC, Gregory S, Hegarty B, McDonald J, Loke S-K (2014). Experimental study on consumer-technology supported authentic immersion in virtual environments for education and vocational training. Rhetoric and reality: critical perspectives on educational technology. Proceedings ascilite Dunedin.

[CR28] Schreiber W (2008). Ein Kompetenz-Strukturmodell historischen Denkens. Zeitschrift für Pädagogik.

[CR29] Schulministerium NRW (2021, September 6). Ministerin Gebauer: Virtual Reality gibt der Digitalisierung einen neuen Schub. Pilotprojekt „Virtual Reality in der Lehrerausbildung“ wird ausgeweitet. [Virtual reality boosts digitization. Pilot project “Virtual Reality in teacher training” is being expanded]. https://www.schulministerium.nrw/presse/pressemitteilungen/ministerin-gebauer-virtual-reality-gibt-der-digitalisierung-einen-neuen. Accessed 25 July 2022.

[CR30] Schuster C, Stebner F, Leutner D, Wirth J (2020). Transfer of metacognitive skills in self-regulated learning: an experimental training study. Metacognition and Learning.

[CR31] Schwonke R, Renkl A, Krieg C, Wittwer J, Aleven V, Salden R (2009). The worked-example effect: not an artefact of lousy control conditions. Computers in human behavior.

[CR32] Shaffer D, Ruis A, Lang C, Siemens G, Wise A, Gašević D (2017). Epistemic network analysis: a worked example of theory-based learning analytics. The handbook of learning analytics.

[CR33] Shaffer DW, Collier W, Ruis AR (2016). A tutorial on epistemic network analysis: analyzing the structure of connections in cognitive, social, and interaction data. Journal of Learning Analytics.

[CR34] Stebner F, Schiffauer S, Schmeck A, Schuster C, Leutner D, Wirth J (2015). Selbstreguliertes Lernen in den Naturwissenschaften. Praxismaterial für die 5. Und 6. Jahrgangsstufe [Self-regulated learning in the natural sciences. Practical material for the 5th and 6th grade].

[CR35] Taranilla VR, Cózar-Gutiérrez R, González-Calero JA, López CI (2019). Strolling through a city of the roman empire: an analysis of the potential of virtual reality to teach history in primary education. Interactive Learning Environments.

[CR36] Teuscher G (2006). Filmanalyse. [film analysis]. Praxis Geschichte.

[CR37] Theobald M (2021). Self-regulated learning training programs enhance university students’ academic performance, self-regulated learning strategies, and motivation: a meta-analysis. Contemporary Educational Psychology.

[CR39] Waldis M, Hodel J, Fink N (2012). Lernaufgaben im Geschichtsunterricht und ihr Potential zur Förderung historischer Kompetenzen. [Learning tasks in history lessons and their potential for promoting historical skills]. Zeitschrift für Didaktik der Gesellschaftswissenschaften.

[CR40] Weinstein CE, Mayer RE, Wittrock M (1986). The teaching of learning strategies. Handbook of Research on Teaching.

[CR41] Wild KP, Schiefele U (1994). Lernstrategien im Studium: Ergebnisse zur Faktorenstruktur und Reliabilität eines neuen Fragebogens. [Learning strategies in college: results on the factor structure and reliability of a new questionnaire]. Zeitschrift für Différentielle und Diagnostische Psychologie.

[CR45] Yek S, Lewers E, Nachtigall V, Bunnenberg C, Rummel N, Chinn C, Tan E, Chan C, Kali Y (2022). Promoting cognitive strategies for processing 360° videos in history learning contexts. International collaboration toward educational innovation for all: overarching research, development, and practices, 16th International Conference of the Learning Sciences (ICLS) 2022.

[CR42] Yildirim G, Elban M, Yildirim S (2018). Analysis of use of virtual reality technologies in history education: a case study. Asian Journal of Education and Training.

[CR43] Zheng L (2016). The effectiveness of self-regulated learning scaffolds on academic performance in computer-based learning environments: a meta-analysis. Asia Pacific Education Review.

[CR44] Zimmermann BJ, Boekaerts M, Pintrich PR, Zeidner M (2000). Attaining self-regulation. A social cognitive perspective. Handbook of Self-Regulation.

